# Special Issue: Advances in Thermal Spray Technology

**DOI:** 10.3390/ma13163521

**Published:** 2020-08-10

**Authors:** Shrikant Joshi

**Affiliations:** Department of Engineering Science, University West, 46132 Trollhättan, Sweden; shrikant.joshi@hv.se

Coatings deposited utilizing different thermal spray variants have been widely used for diverse industrial applications. Today, various coating techniques belonging to the thermal spray family and spanning a vast cost–quality range have been embraced by the industry to either extend the longevity of components or enhance their performance, especially when these parts routinely operate in harsh conditions. The current state-of-the-art route for depositing the ceramic coatings is usually atmospheric plasma spraying (APS), while metallic and intermetallic coatings are sprayed by high-velocity oxy-fuel (HVOF) methods. Between them, the above spray methods and the vast portfolio of commercially available spray grade powders are capable of providing coatings that can combat premature surface degradation when industrial components are exposed to wear, corrosion, oxidation, high thermal load, etc. The immense versatility of the technique has already led to its numerous industrial uses, ranging from the advanced gas turbine requirements to the relatively more mundane needs of sectors such as textile, mining, pulp and paper and petrochemical sectors.

However, efforts continue to explore new potential applications to further expand this envelope. Two of the papers in this Special Issue focusing on impact wear [1] and tribocorrosion properties [2] of sprayed coatings, and another that seeks to augment mechanical properties via plasma spray deposition of multi-constituent amorphous coatings [3] are motivated by the above. Another paper explores novel surface designs to develop thermally sprayed icephobic coatings [4]. With the emergence of new engineering materials such as composites, there is also an interest in implementing thermal spray approaches for imparting them suitable protection, as exemplified by the contribution focusing on ZrC barrier coatings deposited on SiC-coated carbon/carbon composites by vacuum plasma spraying [5]. Post-treatment of thermal sprayed coatings by adopting approaches such as shot peening and laser remelting has also been a subject of considerable academic research. As a complement to such efforts, one of the papers deals with gas nitriding of HVOF-sprayed AISI 316L low-carbon austenitic stainless steel coatings [6].

Traditionally, thermal sprayed coatings have been realized employing powder feedstock, with the particle size typically being in the 10–100 µm range, with the lower end of this range being preferred for high melting point materials such as ceramics. Use of such feedstock, now commercially available for an exhaustive spectrum of material chemistries, results in splat sizes that are several tens of microns and consequently in coarse-structured coatings. However, there is growing interest in realizing fine-structured coatings using submicron and nanosized powders that can potentially yield enhanced functional performance. Such a feedstock injection methodology constitutes the basis for Suspension Plasma Spraying (SPS), which has been found capable of producing coatings with tailored microstructures, including the extremely porous to the very dense, vertically cracked, columnar, etc., and thus are not easily realizable when using a typical spray-grade powder feedstock. With the above approach providing a convenient pathway to deposit fine-structured coatings, thermal spraying with suspensions is perhaps the next frontier. 

A vast majority of interest in SPS has hitherto been driven by the excitement of obtaining columnar thermal barrier coatings (TBCs). One of the papers in this Special Issue investigates SPS-derived double-layered Gadolinium Zirconate/Yttria-Stabilized Zirconia (YSZ) coatings [7]. The rapidly growing interest in this method is apparent from contributions that extend use of SPS to other materials such as oxides [8] and carbides [9], as well as to other high-velocity non-plasma spray processes [8,10]. 

It is also relevant to point out that the advent of axial injection capable plasma spray systems is a potential game-changer for use of liquid feedstock in the form of suspensions or solution precursors. This is by virtue of the fact that axial feeding enables far more intimate contact between the liquid feedstock and the plasma plume to facilitate thermal energy transfer and enable effective utilization of plasma energy. This advantage, which manifests in the form of higher throughputs, longer stand-off distances etc., has been harnessed in a couple of the above-mentioned studies [7,9]. The favourable thermal energy transport between the plasma plume and the suspension feedstock has also encouraged deployment of “hybrid” powder-suspension feedstocks to achieve unique coatings microstructures. One of the papers investigates the performance of such a hybrid powder-suspension sprayed Al_2_O_3_—YSZ coating in bovine serum solution [11].

Admittedly, it was not possible to include in this Special Issue other key areas that, too, continue to play a crucial role in the continued development of thermal spraying. These include, for example, evolution of new torch designs, advanced characterization of coatings, novel approaches for in-flight diagnostics and modelling of coating formation. The use of artificial intelligence/machine learning and data-driven modelling approaches, as illustrated in one of the papers [12], is also destined to play an important role in the future as thermal spray expands into new application domains such as additive manufacturing. Perhaps these can be the focus of a subsequent Special Issue.
